# Training surgeons to optimize communication and symptom management in patients with life-limiting conditions: systematic review

**DOI:** 10.1093/bjsopen/zrad015

**Published:** 2023-04-25

**Authors:** Benjamin E Zucker, Lorna Leandro, Karen Forbes, Jane M Blazeby, Charlotte Chamberlain

**Affiliations:** Population Health Sciences, University of Bristol, Bristol, UK; Division of Surgery, University Hospitals Bristol NHS Foundation Trust, Bristol, UK; Population Health Sciences, University of Bristol, Bristol, UK; Division of Surgery, University Hospitals Bristol NHS Foundation Trust, Bristol, UK; Population Health Sciences, University of Bristol, Bristol, UK; Population Health Sciences, University of Bristol, Bristol, UK; NIHR Biomedical Research Centre, University Hospitals Bristol and Weston NHS Foundation Trust and the University of Bristol, Bristol, UK; Population Health Sciences, University of Bristol, Bristol, UK; Department of Palliative Care, University Hospitals Bristol NHS Foundation Trust, Bristol, UK

## Abstract

**Background:**

Surgeons routinely care for patients with life-limiting illness, requiring communication and symptom management skills supported by appropriate training. The objective of this study was to appraise and synthesize studies that assessed surgeon-directed training interventions that aimed to optimize communication and symptom management for patients with life-limiting illness.

**Methods:**

A PRISMA-concordant systematic review was undertaken. MEDLINE, Embase, Allied and Complementary Medicine Database (AMED), and the Cochrane Central Register of Controlled Trials were searched from inception until October 2022 for studies reporting on the evaluation of surgeon-training interventions intending to improve surgeons’ communication or symptom management of patients with life-limiting disease. Data on the design, trainer and patient participants, and the intervention were extracted. Risk of bias was assessed.

**Results:**

Of 7794 articles, 46 were included. Most studies employed a before–after approach (29 studies) and nine included control groups with five being randomized studies. General surgery was the most frequently included sub-specialty (22 studies). Trainers were described in 25 of 46 studies. Most training interventions aimed to improve communication skills (45 studies) and 13 different training interventions were described. Eight studies reported a measurable improvement in patient care, such as increased documentation of advance care discussions. Most study outcomes focused on surgeons’ knowledge (12 studies), skills (21 studies), and confidence/comfort (18 studies) in palliative communication skills. Studies had a high risk of bias.

**Conclusion:**

Whilst interventions exist to improve the training of surgeons managing patients with life-threatening conditions, evidence is limited, and studies measure the direct impact on patient care insufficiently. Improved research is needed to lead to better methods for training surgeons to benefit patients.

## Teaser

Surgeons routinely care for patients with life-limiting illness, requiring communication and symptom management skills supported by appropriate training. This systematic review found that palliative care training interventions objectively and subjectively improve surgeons’ assessed skill, knowledge, and confidence in palliative communication and symptom management. However, there is insufficient focus on patient outcomes as evidence of the impact of palliative care training.

## Introduction

Surgeons have critical decisions to make about patients’ care that frequently balance the risk of death or disability with potential cure or alleviation of symptom burden. In a study of surgical patients receiving high-risk surgical procedures in the USA, between 15.2 and 24.0 per cent of patients died within 90 days of their procedure^1^. Reports from the USA show that up to one-third of elderly Medicare beneficiaries have an inpatient surgical procedure during their final year of life^2^. Meanwhile, one in five surgical inpatients in the UK is in their last year of life^3^. The *Lancet* Commission on the Value of Death highlights the overmedicalization of those reaching the end of their life, characterized by aggressive treatments and unnecessary suffering. It recommends improved death literacy for surgeons, among other health professionals, as an essential step to rebalancing the care of patients with life-threatening or life-limiting illness^4^. Symptom management and shared decision-making are key to end-of-life care.

Quality shared decision-making in surgery is associated with greater patient satisfaction, increased patient knowledge, and reduced anxiety^5^. Surgical best-practice guidelines in the UK and USA emphasize the importance of generalist palliative care skills in communication with and care of patients who are seriously ill^6-8^. These generalist palliative care skills include both palliative communication skills and skills in symptom management. There is a wealth of literature indicating improved patient and carer outcomes with the involvement of palliative care in the care of patients with life-limiting and life-threatening disease^1,9,10^. Therefore, it is unsurprising that there has been a growing recognition of the importance of palliative care training for surgeons, which has increased in the USA from 16 per cent (2005) to 80 per cent (2018)^11,12^. There are several different methods for training surgeons to achieve improved palliative care skills and shared decision-making, although it is uncertain which is most effective for patient benefit^13^. This study appraised and synthesized studies that assessed surgeon-directed training interventions that aimed to optimize communication and symptom management for patients with life-threatening illness (palliative care skills). The objective of this study was to describe the training interventions and evaluate the outcome measures used in these studies, particularly the use of patient-related outcomes, and understand the efficacy of these studies with relation to outcome measures, including surgeon confidence, knowledge, skill, and comfort.

## Methods

The systematic review was conducted in accordance with PRISMA guidelines^14^ and registered with the International Prospective Register of Systematic Reviews (CRD42020207532).

### Search strategy

OVID SP versions of MEDLINE, Embase, Allied and Complementary Medicine Database (AMED), and the Cochrane Central Register of Controlled Trials were interrogated from time of inception until August 2020, with the search updated in October 2022. The search strategy used a combination of medical subject headings (MeSH) and free-text terms to identify papers that described surgeon-training interventions as below. The search strategy was developed by a palliative care physician (C.C.), surgeon (J.M.B.), and junior doctor (B.E.Z.) with advice from a medical librarian (*Appendix S1*). The search was supplemented by interrogation of reference lists of included studies. Relevant reviews and meta-analyses were screened for studies of interest.

### Included studies

Two reviewers independently undertook study identification through title and abstract screening. Included papers are those that described the evaluation of surgeon-training interventions, defined as any intervention intending to improve surgeons’ communication or symptom management of patients with severe, life-threatening, or life-limiting disease. Included in this study are mixed-population studies where surgeons were explicitly stated to be included. Key terms were used to identify palliative care focus in studies for inclusion. These included: palliative care, prognosis, goal orientated, goal concordant, advance care plan (ACP), end of life (EoL), serious illness, life-limiting illness, life-threatening, terminal care, and ‘breaking bad news’. Intervention studies that reported the development of an intervention but did not assess its impact (that is that did not report clinician or patient outcomes) were excluded. Disagreements were resolved by discussion with the third reviewer/senior author. Eligible study designs included: RCTs, before–after studies, correlational studies, studies utilizing a single-arm experimental design, and quality improvement projects. Non-interventional studies, studies with insufficient primary data (opinion pieces, congress abstracts), qualitative studies, and non-English language studies were excluded. Where studies included some embedded qualitative analysis, qualitative results were not included in the narrative synthesis. Studies that focused on generic communication skills without any palliative care content were excluded. In addition, studies conducted with paediatric/obstetric surgeons were excluded due to the inherent difference in the surgeon–patient interactions.

### Data extraction

Data extraction was undertaken by two reviewers independently. Data were recorded electronically in an Excel spreadsheet. Data relating to a description of the study included: author, year of publication, journal, study design, funding source, and the country in which the study took place. Data relating to the description of the intervention included: clinician inclusion and exclusion criteria, the sub-specialty of surgeons, patient characteristics, the number and discipline of educators/trainers, content and focus of the intervention (communication and/or symptom management), and the intervention training approach, including the number of approaches used. The results of intervention evaluation that pertained to clinician outcomes (such as change in knowledge or confidence) or patient impact (such as documentation of Do Not Attempt Cardiopulmonary Resuscitation (DNACPR) for patients treated by surgeons who had received the palliative training intervention) were extracted.

### Quality assessment

The quality of reporting of all included studies was assessed using the Evidence Project risk-of-bias tool by two reviewers independently^15^. This tool was chosen due to its applicability to a range of study designs. Studies are not given a summary score of bias, but areas of significant bias within studies are highlighted.

### Analysis

Studies were analysed first according to the focus of the intervention (communication or symptom management) and second according to the intervention outcome: outcomes, termed in this paper as ‘patient outcomes,’ that reflected quality of patient care, such as patient experience surveys, documentation of advance care planning discussions, or length of hospital stay; or studies that focused on surgeon benefit (self-assessed or externally assessed), such as the clinicians’ perceived value of the intervention (confidence, knowledge, and self-reported practice) or objectively perceived changes in communication skills (*Table S1*).

A narrative account of interventions to improve essential generalist palliative care skills in surgeons was created using the framework described by Popay *et al*.^16^. A theory of how the intervention may have worked for surgeons was discussed and a preliminary synthesis of findings prepared and discussed with the research team. The robustness of the synthesis was tested by comparing the findings against relationships within the data.

## Results

### Results of the search

**Fig. 1:**
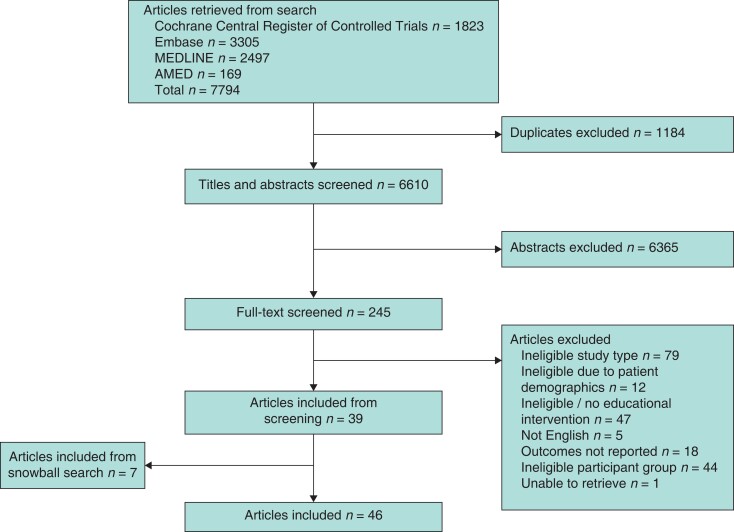
PRISMA diagram.

Of 7794 screened articles, 46 were included in the final analyses (*Fig. 1*)^13,17-61^.

AMED, Allied and Complementary Medicine Database

### Included studies

#### Study description

Most studies employed a before–after approach (29 studies). Nine studies included control arms, of which five were RCTs. Five studies employed a single-arm experimental design. Two studies utilized an interrupted time-series design. Most studies took place in the USA (34 studies), four took place in Canada, three took place in Japan, two took place in Israel, two took place in Belgium, and one took place in Pakistan (*Table S1*).

#### Intervention description

The reported detail of the training intervention varied between studies. Studies involved between three and 345 surgeons (median = 31). Two studies included fewer than ten surgeons^21,48^. One was a teaching programme for juniors rotating through an intensive care unit (ICU), including three surgeons. The other, including eight surgeons, was a pilot of a grand round and objective structured clinical examination (OSCE) of leading a family conference in an ICU. Thirty studies described the surgeons’ specialties. Frequently included specialties were general surgery (22 studies), gynaecology (6 studies), cardiothoracics (5 studies), and orthopaedics (5 studies). Fifteen of the included studies were mixed groups of surgeons and non-surgical clinicians.

Patient characteristics were described in nine studies^28,37,40,43,46,50,58-60^. These patients were described as having specifically surgical issues in seven of these studies^28,40,43,50,58-60^.

Individuals delivering the training intervention were described in 25 of 46 studies with trainers’ descriptions frequently referencing expertise in education, communication, or palliative care^13,19,22-25,28,31-33,35,36,38,40-43,45,50,52,55,56,59-61^.

The focus and therefore the content of the training interventions varied. Study focus included a focus on improving knowledge, skills, and/or behaviour. Some studies aimed to deliver all three aims, for instance, addressing communication knowledge and skills by supporting patients to describe their advance care wishes (advance directives/advance decision to refuse treatment (ADRT)) and having these appropriately documented.

Most interventions were delivered face-to-face in lecture and/or small-group format with facilitators present (33 of 46)^13,17,20-22,24,26-30,32-36,38,40-42,45,46,49,50,52-59,61^. The majority of studies employed more than one training approach (for example lectures, small-group discussion, simulation). Thirteen different training intervention approaches were described, including lectures/grand rounds, simulation, literature review, e-modules, vignette review, structured debriefing, and simulated patient encounters (*Table S1*). Five studies included an online training component, including e-modules; two were delivered solely online^18,33,39,51,60^. The most common face-to-face modalities included lectures/grand rounds, small-group discussion, role play/simulation, and case-based discussion. Infrequently used modalities included virtual e-modules and journal article review.

### Results of analysis

#### Description of included studies measuring patient outcomes

Nine studies included a measure of patient care after surgical training in palliative communication and symptom management^28,37,40,43,46,50,58-60^. Between one and six patient-specific care measures were included per study with significant inter-study heterogeneity. The most common patient care outcome was documentation of code or resuscitation status, advance directives/ADRT, or goals-of-care (GOC) conversations. Other outcomes included: the number of patients who received surgery, were admitted to ICU, received a palliative care consultation, the patient discharge destination^40^, length of hospital stay (for those patients needing ICU care)^28,59^, pain assessment (based on a notes review)^37^, patient-reported frequency with which doctors provided understandable information^43^, ventilator days, mortality rate, disposition, and frequency of invasive procedures^58,59^. Eight studies reported improvements in some patient care outcomes associated with the intervention. These included: increased likelihood that a physician had a GOC discussion^46^, increased frequency of documentation of both advance directives and code or resuscitation status^50,59,60^, reduced time to GOC conversation^59^, decreased non-beneficial care in an ICU setting^28^, increased patient rating of doctors’ ability to provide understandable explanations^43^, improved shared decision-making^40^ and improved pain scores of patients treated by surgeons who had undertaken a rotation in palliative care^37^, and a decrease in palliative care consultation^59^.

Several studies described no improvement in specific patient outcomes related to palliative care training interventions. Amen *et al*.^59^ implemented a 4-week multi-modal training programme for residents rotating through the surgical ICU and found an increase in ACP documentation and a reduction in ICU length of stay. However, they noted no change in mortality rate or the percentage of patients discharged in a persistent vegetative state^59^. A further study found no difference in the intensity of received treatment between patient groups pre- and post-intervention where trauma Attendings received training regarding the ‘best case/worse case’ (Bc/WC) framework^58^, a communication approach to help support surgeons have goal-orientated patient consultations.

Taylor *et al*.^40^ also evaluated the introduction of the Bc/WC framework. This approach demonstrated mixed findings. There was improved shared decision-making using the OPTION 5 score (an observer measure of shared decision-making based on a 100 point scale) with an improvement in median score from 41 pre-intervention to 74 after training (intraclass correlation was 0.80 (95 per cent c.i. 0.64 to 0.90)). Patients seen by surgeons after the training intervention more frequently received the proposed surgery (42 per cent before the intervention *versus* 50 per cent after the intervention). However, patients seen by surgeons after the intervention were less frequently discharged home (50 per cent before the intervention *versus* 20 per cent after the intervention) or to a hospice (17 per cent before the intervention *versus* 5 per cent after the intervention) and were more frequently discharged to skilled nursing facilities (25 per cent before the intervention *versus* 45 per cent after the intervention)—although the intention of discharge (for example rehabilitation) was not stated.

#### Description of communication skill studies

Forty-five studies reported palliative communication skill outcomes^13,17-36,38-61^. Heterogeneous outcomes included: clinician perceived value of the intervention (25 studies), objectively assessed communication skills—assessed by a mixture of faculty, family, patients, or simulated patients (21 studies), self-assessed confidence, comfort, or competence (18 studies), objectively assessed knowledge (12 studies), self-reported practice improvement (7 studies), patient outcomes (7 studies), and ‘other’ (17 studies)—including perception of palliative care (measured by questionnaire), reported difficulties in practice, and participant attitudes regarding physician–patient communication.

##### Perceived value of the intervention

The perceived value of the intervention was measured in 25 studies and as such was the most commonly reported outcome measure of the 45 studies^13,17,21,23-25,30-33,35,36,39,41-46,48,49,51,52,56,58^. It was principally measured using questionnaires and consistently rated as being valued highly.

##### Communication skills

Of the 21^19-23,26,27,30,32,33,39-41,43,44,51,53,56,57,58,61^ studies objectively assessing communication skills (assessed by faculty, simulated patients, or patients), eight^20-23,32,41,44,51^ studies did not include either a pre-test or comparison/control group for this outcome measure. Four studies used both a control/comparison group and a pre- and post-intervention design^39,53,57,61^. Three of these found that an intensive communication skill training course significantly improved the palliative communication skills of clinicians^53,57,61^. One study compared an intervention group with a comparison group post-intervention, but not to baseline scores—this study found no difference between groups^19^. Of eight^26,27,30,33,40,43,56,58^ studies using a before–after design with no control group, six showed significant improvement after the intervention^26,30,40,43,56,59^ compared with clinicians’ baseline scores. Risk of bias was generally high across these studies due to the paucity of studies utilizing control groups and the poor follow-up rates of the before–after studies, of which only three had a follow-up rate of greater than or equal to 80 per cent^27,30,40^.

##### Self-reported confidence, comfort, or competence

Clinicians’ self-assessed confidence, comfort, or competence relating to communication skills was recorded in 18 studies. Only one study^34^ included pre- and post-intervention tests and a comparison group to examine self-assessed confidence, comfort, or competence in generalist palliative communication skills. This study showed no difference between intervention and control groups. One study used a control group and post-pre assessment, with retrospective assessment occurring at the time of post-intervention assessment. This study showed significant improvement in the intervention group, but did not report the results of the control group^61^. Sixteen^13,17,19,20,24,31,33,35,36,42-44,47,48,51,60^ studies tested this outcome measure using pre- and post-intervention tests (without a comparison group); of these, 11^13,17,24,31,35,36,42,43,47,48,60^ showed increased self-reported confidence, comfort, or competence measures. However, the lack of control groups used in the evaluation of this outcome measure is indicative of a high risk of bias in these results.

##### Objective knowledge

Twelve studies^17-19,22,29,31,35,38,42,45,54,55^ measured change in knowledge of communication strategies and context, of which three^19,29,45^ showed significant improvement when compared with a control group. A further seven studies^18,22,35,38,42,54,55^, of which three had a follow-up rate of greater than 80 per cent^22,35,54^, showed improvement after a palliative care intervention using pre- and post-intervention tests.

##### Self-reported practice

Study clinician-participants’ self-reported practice improved across all seven^20,29,36,41,49,50,54^ studies that measured this outcome, with one study showing this compared with a control group^29^ and another reporting sustained improvement 6 months post-intervention^20^. However, the evaluation of this outcome is at risk of bias due to the few control groups used in its evaluation.

#### Description of symptom management studies

Among the seven^19,29,34,37,38,54,55^ studies investigating palliative symptom management, six measured knowledge acquisition^19,29,34,38,54,55^. All six studies showed that some knowledge domains (for example management of cancer pain, opioid side effects, delirium, and symptom markers of poor end-of-life care) improved after the training intervention. Only two of these showed improvement compared with a control group^29,34^. Three^19,34,37^ of the seven symptom management studies measured self-assessed confidence, of which one compared this with a control group^34^, and all showed improvement associated with the intervention. However, the lack of control groups in most studies suggests a high risk of bias in these studies.

Both studies that reported on self-reported practice relating to care of the dying patient (including pain and dyspnoea) found an improvement in self-reported practice scores after the intervention, with only one showing this compared with a control group^29,54^.

### Quality assessment

The Evidence Project risk-of-bias tool checklist is presented in *Table S2*. Twenty-two studies reached the Evidence Project risk-of-bias tool predefined follow-up rate of 80 per cent in at least one domain (for example in the knowledge test). Forty-five studies did not select clinician-participants randomly for assessment.

## Discussion

This systematic review found 46 studies evaluating surgeon-directed palliative care training. Of these, nine studies measured the impact of surgeon-directed training on patient care outcomes, with eight showing improvement in some patient care outcome measures, such as shared decision-making. Of these nine studies, three were published since 2021. The remainder focused on benefits to surgeons. Overall, included studies showed that surgeons’ skill, knowledge, confidence, and comfort with palliative communication and symptom management improved after the training interventions. However, the subjective nature of some outcomes, such as self-perceived confidence and comfort, and the frequent absence of control groups, limits the extent to which they can be considered evidence for the success of an intervention. This is especially true given the conflicting literature on the relationship between clinicians’ confidence and competence^62,63^. Therefore, while there is some mixed evidence that surgeon-directed palliative care training improves patient care and perceived knowledge and skills amongst surgeons, the latter cannot be extrapolated to demonstrate sustained improvement in patient care conclusively. This emphasizes the need for more research in this area.

This review echoes concerns raised in a 2017 systematic review around training surgeons and anaesthesiologists in end-of-life conversations, describing flawed study methodology, such as an absence of control groups, and inadequate patient outcome measures^64^. Compared with previous reviews, the current review has identified some progress in the field, with studies that report specifically on communication in the perioperative interval^40^ and communication of poor prognosis and unexpected death in the trauma bay^51^, which previously has been found lacking^65^.

It has been suggested that surgeons have reduced responsiveness to emotional cues and increased focus on medical details^66,67^. However, whether training surgeons to redress the balance to have more effective goal-concordant discussions is the most effective means to improve outcomes for patients with life-limiting illness in surgical settings is not known. There is evidence that multidisciplinary working between surgeons, palliative care physicians, and geriatricians is associated with reduced length of stay, healthcare costs, and mortality rate^68,69^. However, there is no evidence comparing training surgeons in palliative care skills with other organizational levers that increase multidisciplinary working in the perioperative interval.

Surgical referrals to specialist palliative care have increased over time. This is attributed to the introduction of greater palliative care exposure in training and a culture change stemming from publications, such as the American College of Surgeons’ Statement of Principles of Palliative Care^12^, and the UK Royal College of Surgeons’ Caring for Patients Nearing the End of Life^6^. However, a number of studies still describe inadequate inclusion of palliative care in surgical curricula and identify gaps in surgical training around end-of-life conversations and prognostication in particular; therefore, this review is timely and important^21-23^.

The strengths of this review include the completeness of the search strategy and inclusion criteria. No other systematic review in the field has included interventional studies aimed at improving palliative communication skills and symptom management for surgeons. The internal validity of this review was strengthened by dual and independent abstract screening, full-text screening, data extraction, and quality assessment. A limitation is the non-inclusion of studies reporting on the development of training interventions, as key interventions may consequently have been missed. Non-controlled studies were included in this study due to the frequency of surgeon perception, experience, or change in practice as a study outcome measure.

A limitation of the data is the inability to carry out a meta-analysis due to the heterogeneity of interventions, outcome measures, and methods of evaluation. Unfortunately, the range of multi-modal intervention approaches, combined with a variety of outcome measures, mean it is impossible to identify the most effective training intervention.

The measurement of patient care as an assessment of training quality is becoming more widespread^70,71^. It is clear this evolution would also benefit studies of surgical postgraduate training. Many of the included studies that addressed patient care outcomes relied on routine data, such as length of stay, or documentation of GOC discussions. The greater use of patient experience and palliative care-specific outcome measures, such as the palliative care outcome scale (POS)^72^, which consider the physical, psychosocial, and spiritual domains of care quality, would be beneficial.

Important and recurring limitations of included studies included single-centre, small sample-size studies with unvalidated, non-systematic outcome assessment that did not account for a maturation effect or sustained change over time. Other important limitations to note are the infrequency with which control or comparison groups were used, the lack of randomization, and the paucity of studies reporting patient outcomes. All of these limitations are common in the literature around the effectiveness of postgraduate training interventions^73^.

This study varied from the published protocol in the exclusion of studies reporting on anaesthetists and intensivists as a participant population in the absence of surgical participants. This was due to the large number of studies retrieved and the consequent difficulty in synthesizing and comparing results. In addition, qualitative results were not included due to the significant heterogeneity in methods utilized and outcomes measured.

Future research in postgraduate palliative care training would benefit from universally agreed patient outcome measures. While the use of surrogate outcomes in training interventions, such as clinician confidence or clinician-perceived value of the intervention, has advantages (reduced cost, simpler and less time-consuming ethical approval), their continued use requires demonstration that they reflect a pathway to improved patient care. Reporting of postgraduate training interventions can be improved using a bespoke statement, such as the guideline for reporting evidence-based practice educational interventions and teaching (GREET) statement^74^ or the criteria for describing and evaluating training interventions in healthcare professions (CRE-DEPTH)^75^. Although the GREET statement is not specific to palliative care postgraduate training, its use would significantly improve reporting, and, where consulted at intervention development, would likely improve the quality of studies performed^76^. Finally, underlying epidemiological principles clearly point to the need to improve intervention design with the use of randomization and control arms for training interventions and systematic outcome reporting methods.

This study provides the most complete overview of attempts to improve palliative care skills in surgeons to date. Palliative care training interventions objectively and subjectively improve surgeons’ assessed skill, knowledge, and confidence in palliative communication and symptom management. Nevertheless, there is insufficient focus on patient outcomes as evidence of the impact of palliative care training, and heterogeneity of patient outcome measures confounds synthesis of the study findings. However, of the studies published since 2021, three of five studies included use of patient outcome measures, indicating potential progress in this field of research. Greater implementation of standardized reporting statements in studies of postgraduate palliative care training would improve the conduct and reporting of research in this field.

## Supplementary Material

zrad015_Supplementary_Data

## Data Availability

Data will be provided by the corresponding author upon reasonable request.

## References

[1] Yefimova M , Aslakson RA, Yang L, Garcia A, Boothroyd D, Gale RC et al Palliative care and end-of-life outcomes following high-risk surgery. JAMA Surg 2020;155:138–146 31895424 10.1001/jamasurg.2019.5083 PMC6990868

[2] Kwok AC , Semel ME, Lipsitz SR, Bader AM, Barnato AE, Gawande AA et al The intensity and variation of surgical care at the end of life: a retrospective cohort study. Lancet 2011;378:1408–1413 21982520 10.1016/S0140-6736(11)61268-3

[3] Clark D , Schofield L, Graham FM, Isles C, Gott M, Jarlbaek L. Likelihood of death within one year among a national cohort of hospital inpatients in Scotland. J Pain Symptom Manage 2016;52:e2–e4 10.1016/j.jpainsymman.2016.05.007 27262261

[4] Sallnow L , Smith R, Ahmedzai SH, Bhadelia A, Chamberlain C, Cong Y et al Report of the *Lancet* Commission on the Value of Death: bringing death back into life. Lancet 2022;399:837–884 35114146 10.1016/S0140-6736(21)02314-X PMC8803389

[5] Niburski K , Guadagno E, Abbasgholizadeh-Rahimi S, Poenaru D. Shared decision making in surgery: a meta-analysis of existing literature. Patient 2020;13:667–681 32880820 10.1007/s40271-020-00443-6

[6] Royal College of Surgeons of England . Caring for Patients Nearing the End of Life. https://www.rcseng.ac.uk/standards-and-research/standards-and-guidance/good-practice-guides/end-of-life-care/ (accessed February 2021)

[7] Mack JW , Weeks JC, Wright AA, Block SD, Prigerson HG. End-of-life discussions, goal attainment, and distress at the end of life: predictors and outcomes of receipt of care consistent with preferences. J Clin Oncol 2010;28:1203–1208 20124172 10.1200/JCO.2009.25.4672 PMC2834470

[8] Scally CP , Robinson K, Blumenthaler AN, Bruera E, Badgwell BD. Identifying core principles of palliative care consultation in surgical patients and potential knowledge gaps for surgeons. J Am Coll Surg 2020;231:179–185 32311465 10.1016/j.jamcollsurg.2020.03.036 PMC7714396

[9] El-Jawahri A , Greer JA, Temel JS. Does palliative care improve outcomes for patients with incurable illness? A review of the evidence. J Support Oncol 2011;9:87–94 21702398 10.1016/j.suponc.2011.03.003

[10] Zimmermann C , Swami N, Krzyzanowska M, Hannon B, Leighl N, Oza M et al Early palliative care for patients with advanced cancer: a cluster-randomised controlled trial. Lancet 2014;383:1721–1730 24559581 10.1016/S0140-6736(13)62416-2

[11] Galante JM , Bowles TL, Khatri VP, Schneider PD, Goodnight JE Jr, Bold RJ. Experience and attitudes of surgeons toward palliation in cancer. Arch Surg 2005;140:873–878 16172296 10.1001/archsurg.140.9.873

[12] Bateni SB , Canter RJ, Meyers FJ, Galante JM, Bold RJ. Palliative care training and decision-making for patients with advanced cancer: a comparison of surgeons and medical physicians. Surgery 2018. doi: 10.1016/j.surg.2018.01.021 PMC6204113 29709369

[13] Weill SR , Layden AJ, Nabozny MJ, Leahy J, Claxton R, Zelenski AB et al Applying VitalTalk™ techniques to best case/worst case training to increase scalability and improve surgeon confidence in shared decision-making. J Surg Educ 2022;79:983–992 35246401 10.1016/j.jsurg.2022.01.012

[14] Moher D , Liberati A, Tetzlaff J, Altman DG. Preferred reporting items for systematic reviews and meta-analyses: the PRISMA statement. BMJ 2009;339:b2535 19622551 10.1136/bmj.b2535 PMC2714657

[15] Kennedy CE , Fonner VA, Armstrong KA, Denison JA, Yeh PT, O’Reilly KR et al The Evidence Project risk of bias tool: assessing study rigor for both randomized and non-randomized intervention studies. Syst Rev 2019;8:3 30606262 10.1186/s13643-018-0925-0 PMC6317181

[16] Popay J , Roberts H, Sowden A, Petticrew M, Arai L, Rodgers M et al Guidance on the conduct of narrative synthesis in systematic reviews. ESRC Methods Programme 2006. https://www.lancaster.ac.uk/media/lancaster-university/content-assets/documents/fhm/dhr/chir/NSsynthesisguidanceVersion1-April2006.pdf (accessed August 2020)

[17] Angelos P , DaRosa DA, Derossis AM, Kim B. Medical ethics curriculum for surgical residents: results of a pilot project. Surgery 1999;126:701–705 10520918

[18] Bergman J , Lorenz KA, Ballon-Landa E, Kwan L, Lerman SE, Saigal CS et al A scalable web-based module for improving surgical and medical practitioner knowledge and attitudes about palliative and end-of-life care. J Palliat Med 2015;18:415–420 25748832 10.1089/jpm.2014.0349

[19] Bradley CT , Webb TP, Schmitz CC, Chipman JG, Brasel KJ. Structured teaching versus experiential learning of palliative care for surgical residents. Am J Surg 2010;200:542–547 20538256 10.1016/j.amjsurg.2009.12.014

[20] Chesney T , Devon K. Training surgical residents to use a framework to promote shared decision-making for patients with poor prognosis experiencing surgical emergencies. Can J Surg 2018;61:114–120 29582747 10.1503/cjs.011317 PMC5866147

[21] Chipman JG , Beilman GJ, Schmitz CC, Seatter SC. Development and pilot testing of an OSCE for difficult conversations in surgical intensive care. J Surg Educ 2007;64:79–87 17462207 10.1016/j.jsurg.2006.11.001

[22] Fanous A , Rappaport J, Young M, Park YS, Manoukian J, Nguyen LHP. A longitudinal simulation-based ethical-legal curriculum for otolaryngology residents. Laryngoscope 2017;127:2501–2509 28850677 10.1002/lary.26551

[23] Gettman MT , Karnes RJ, Arnold JJ, Klipfel JM, Vierstraete HT, Johnson ME et al Urology resident training with an unexpected patient death scenario: experiential learning with high fidelity simulation. J Urol 2008;180:283–288 18499174 10.1016/j.juro.2008.03.042

[24] Haglund MM , Rudd M, Nagler A, Prose NS. Difficult conversations: a national course for neurosurgery residents in physician-patient communication. J Surg Educ 2015;72:394–401 25687955 10.1016/j.jsurg.2014.11.014

[25] Harnof S , Hadani M, Ziv A, Berkenstadt H. Simulation-based interpersonal communication skills training for neurosurgical residents. Isr Med Assoc J 2013;15:489–492 24340839

[26] Hochberg MS , Kalet A, Zabar S, Kachur E, Gillespie C, Berman RS. Can professionalism be taught? Encouraging evidence. Am J Surg 2010;199:86–93 20103071 10.1016/j.amjsurg.2009.10.002

[27] Hochberg MS , Berman RS, Kalet AL, Zabar SR, Gillespie C, Pachter HL. The professionalism curriculum as a cultural change agent in surgical residency education. Am J Surg 2012;203:14–20 21983000 10.1016/j.amjsurg.2011.05.007

[28] Holloran SD , Starkey GW, Burke PA, Steele G Jr, Forse RA. An educational intervention in the surgical intensive care unit to improve ethical decisions. Surgery 1995;118:294–298 7638746 10.1016/s0039-6060(05)80337-x

[29] Inoue A , Yamaguchi T, Tanaka K, Sakashita A, Aoe K, Seki N et al Benefits of a nationwide palliative care education program on lung cancer physicians. Intern Med 2019;58:1399–1403 30713293 10.2169/internalmedicine.0872-18 PMC6548920

[30] Jameel A , Noor SM, Ayub S, Ali SS, Park YS, Tekian A. Feasibility, relevance and effectiveness of teaching and assessment of ethical status and communication skills as attributes of professionalism. J Pak Med Assoc 2015;65:721–726 26160080

[31] Klaristenfeld DD , Harrington DT, Miner TJ. Teaching palliative care and end-of-life issues: a core curriculum for surgical residents. Ann Surg Oncol 2007;14:1801–1806 17342567 10.1245/s10434-006-9324-1

[32] Kruser JM , Taylor LJ, Campbell TC, Zelenski A, Johnson SK, Nabozny MJ et al “Best case/worst case”: training surgeons to use a novel communication tool for high-risk acute surgical problems. J Pain Symptom Manage 2017;53:711–719.e5 28062349 10.1016/j.jpainsymman.2016.11.014 PMC5374034

[33] Margolis B , Blinderman C, de Meritens AB, Chatterjee-Paer S, Ratan RB, Prigerson HG et al Educational intervention to improve code status discussion proficiency among obstetrics and gynecology residents. Am J Hosp Palliat Care 2018;35:724–730 28950726 10.1177/1049909117733436

[34] Mikhael J , Baker L, Downar J. Using a pocket card to improve end-of-life care on internal medicine clinical teaching units: a cluster-randomized controlled trial. J Gen Intern Med 2008;23:1222–1227 18446415 10.1007/s11606-008-0582-4 PMC2517963

[35] Moon MR , Hughes MT, Chen JY, Khaira K, Lipsett P, Carrese JA. Ethics skills laboratory experience for surgery interns. J Surg Educ 2014;71:829–838 25012607 10.1016/j.jsurg.2014.03.010

[36] Nakagawa S , Fischkoff K, Berlin A, Arnell TD, Blinderman CD. Communication skills training for general surgery residents. J Surg Educ 2019;76:1223–1230 31005480 10.1016/j.jsurg.2019.04.001

[37] Oya H , Matoba M, Murakami S, Ohshiro T, Kishino T, Satoh Y et al Mandatory palliative care education for surgical residents: initial focus on teaching pain management. Jpn J Clin Oncol 2013;43:170–175 23275645 10.1093/jjco/hys205 PMC3559015

[38] Raoof M , O’Neill L, Neumayer L, Fain M, Krouse R. Prospective evaluation of surgical palliative care immersion training for general surgery residents. Am J Surg 2017;214:378–383 27908501 10.1016/j.amjsurg.2016.11.032

[39] Schmitz CC , Braman JP, Turner N, Heller S, Radosevich DM, Yan Y et al Learning by (video) example: a randomized study of communication skills training for end-of-life and error disclosure family care conferences. Am J Surg 2016;212:996–1004 27474496 10.1016/j.amjsurg.2016.02.023

[40] Taylor LJ , Nabozny MJ, Steffens NM, Tucholka JL, Brasel KJ, Johnson SK et al A framework to improve surgeon communication in high-stakes surgical decisions: best case/worst case. JAMA Surg 2017;152:531–538 28146230 10.1001/jamasurg.2016.5674 PMC5479749

[41] Taylor LJ , Adkins S, Hoel AW, Hauser J, Suwanabol P, Wood G et al Using implementation science to adapt a training program to assist surgeons with high-stakes communication. J Surg Educ 2019;76:165–173 30626527 10.1016/j.jsurg.2018.05.015

[42] Thirunavukarasu P , Brewster LP, Pecora SM, Hall DE. Educational intervention is effective in improving knowledge and confidence in surgical ethics-a prospective study. Am J Surg 2010;200:665–669 21056150 10.1016/j.amjsurg.2010.08.002

[43] Trickey AW , Newcomb AB, Porrey M, Piscitani F, Wright J, Graling P et al Two-year experience implementing a curriculum to improve residents’ patient-centered communication skills. J Surg Educ 2017;74:e124–e132 28756146 10.1016/j.jsurg.2017.07.014

[44] Wehbe-Janek H , Song J, Shabahang M. An evaluation of the usefulness of the standardized patient methodology in the assessment of surgery residents’ communication skills. J Surg Educ 2011;68:172–177 21481799 10.1016/j.jsurg.2010.12.005

[45] Wenger NS , Liu H, Lieberman JR. Teaching medical ethics to orthopaedic surgery residents. J Bone Joint Surg Am 1998;80:1125–1131 9730121 10.2106/00004623-199808000-00005

[46] Childers JW , Arnold RM. Expanding goals of care conversations across a health system: the mapping the future program. J Pain Symptom Manage 2018;56:637–644 30048766 10.1016/j.jpainsymman.2018.07.013

[47] Harrington AW , Oliveira KD, Lui FY, Maerz LL. Resident education in end-of-life communication and management: assessing comfort level to enhance competence and confidence. J Surg Educ 2020;77:300–308 31780426 10.1016/j.jsurg.2019.11.003

[48] Minor S , Schroder C, Heyland D. Using the intensive care unit to teach end-of-life skills to rotating junior residents. Am J Surg 2009;197:814–819 18789413 10.1016/j.amjsurg.2008.04.015

[49] Brezis M , Lahat Y, Frankel M, Rubinov A, Bohm D, Cohen MJ et al What can we learn from simulation-based training to improve skills for end-of-life care? Insights from a national project in Israel. Isr J Health Policy Res 2017;6:48 29110738 10.1186/s13584-017-0169-9 PMC5674237

[50] Cantu RV , Coe MP, Pober DM, Byock IR. Orthopedic grand rounds can change resident practice. Am J Orthop (Belle Mead NJ) 2013;42:215–219 23710477

[51] Fiorentino M , Mosenthal AC, Bryczkowski S, Lamba S. Teaching residents communication skills around death and dying in the trauma bay. J Palliat Med 2021;24:77–82 32716675 10.1089/jpm.2020.0076

[52] Klingensmith ME . Teaching ethics in surgical training programs using a case-based format. J Surg Educ 2008;65:126–128 18439534 10.1016/j.jsurg.2007.12.001

[53] Liénard A , Merckaert I, Libert Y, Bragard I, Delvaux N, Etienne A-M et al Is it possible to improve residents breaking bad news skills? A andomized study assessing the efficacy of a communication skills training program. Br J Cancer 2010;103:171–177 20628395 10.1038/sj.bjc.6605749 PMC2906741

[54] Yamamoto R , Kizawa Y, Nakazawa Y, Ohde S, Tetsumi S, Miyashita M. Outcome evaluation of the palliative care emphasis program on symptom management and assessment for continuous medical education: nationwide physician education project for primary palliative care in Japan. J Palliat Med 2015;18:45–49 25495030 10.1089/jpm.2014.0122

[55] Pernar LI , Peyre SE, Smink DS, Block SD, Cooper ZR. Feasibility and impact of a case-based palliative care workshop for general surgery residents. J Am Coll Surg 2012;214:231–236 22169003 10.1016/j.jamcollsurg.2011.11.002

[56] Larkin AC , Cahan MA, Whalen G, Hatem D, Starr S, Haley H-L et al Human emotion and response in surgery (HEARS): a simulation-based curriculum for communication skills, systems-based practice, and professionalism in surgical residency training. J Am Coll Surg 2010;211:285–292 20670869 10.1016/j.jamcollsurg.2010.04.004

[57] Merckaert I , Liénard A, Libert Y, Bragard I, Delvaux N, Etienne A-M et al Is it possible to improve the breaking bad news skills of residents when a relative is present? A andomized study. Br J Cancer 2013;109:2507–2514 24129243 10.1038/bjc.2013.615 PMC3833209

[58] Zimmermann CJ , Zelenski AB, Buffington A, Baggett ND, Tucholka JL, Weis HB et al Best case/worst case for the trauma ICU: development and pilot testing of a communication tool for older adults with traumatic injury. J Trauma Acute Care Surg 2021;91:542–551 34039930 10.1097/TA.0000000000003281 PMC8939782

[59] Amen SS , Berndtson AE, Cain J, Onderdonk C, Cochran-Yu M, Gambles Farr S et al Communication and palliation in trauma critical care: impact of trainee education and mentorship. J Surg Res 2021;266:236–244 34029763 10.1016/j.jss.2021.03.005

[60] Kaminski A . Let’s talk about dying: an educational pilot program to improve providers’ competency in end-of-life discussions. Am J Hosp Palliat Care 2022;10499091221127994 10.1177/10499091221127994 36154272

[61] Lockwood BJ , Gustin J, Verbeck N, Rossfeld K, Norton K, Barrett T et al Training to promote empathic communication in graduate medical education: a shared learning intervention in internal medicine and general surgery. Palliative Med Rep 2022;3:26–35 10.1089/pmr.2021.0036 PMC8994435 35415720

[62] Dehmer JJ , Amos KD, Farrell TM, Meyer AA, Newton WP, Meyers MO. Competence and confidence with basic procedural skills: the experience and opinions of fourth-year medical students at a single institution. Acad Med 2013;88:682–687 23524922 10.1097/ACM.0b013e31828b0007

[63] Leopold SS , Morgan HD, Kadel NJ, Gardner GC, Schaad DC, Wolf FM. Impact of educational intervention on confidence and competence in the performance of a simple surgical task. J Bone Joint Surg Am 2005;87:1031–1037 15866966 10.2106/JBJS.D.02434

[64] Bakke KE , Miranda SP, Castillo-Angeles M, Cauley CE, Lilley EJ, Bernacki R et al Training surgeons and anesthesiologists to facilitate end-of-life conversations with patients and families: a systematic review of existing educational models. J Surg Educ 2018;75:702–721 28939306 10.1016/j.jsurg.2017.08.006

[65] Lamba S , Tyrie LS, Bryczkowski S, Nagurka R. Teaching surgery residents the skills to communicate difficult news to patient and family members: a literature review. J Palliat Med 2016;19:101–107 26575251 10.1089/jpm.2015.0292

[66] Levinson W , Gorawara-Bhat R, Lamb J. A study of patient clues and physician responses in primary care and surgical settings. JAMA 2000;284:1021–1027 10944650 10.1001/jama.284.8.1021

[67] Maguire P , Faulkner A. Communicating with cancer patients. BMJ 1988;297:1610 10.1136/bmj.297.6663.1610 PMC1835267 3147098

[68] Centre for Perioperative Care . Impact of Perioperative Care on Healthcare Resource Use. Rapid Research Review; 2020. https://cpoc.org.uk/sites/cpoc/files/documents/2020-09/Impact%20of%20perioperative%20care%20-%20rapid%20review%20FINAL%20-%2009092020MW.pdf (accessed February 2021)

[69] Ernst KF , Hall DE, Schmid KK, Seever G, Lavedan P, Lynch TG et al Surgical palliative care consultations over time in relationship to systemwide frailty screening. JAMA Surg 2014;149:1121–1126 25207603 10.1001/jamasurg.2014.1393 PMC4603652

[70] Tamblyn R , Abrahamowicz M, Dauphinee WD, Hanley JA, Norcini J, Girard N et al Association between licensure examination scores and practice in primary care. JAMA 2002;288:3019–3026 12479767 10.1001/jama.288.23.3019

[71] Bansal N , Simmons KD, Epstein AJ, Morris JB, Kelz RR. Using patient outcomes to evaluate general surgery residency program performance. JAMA Surg 2016;151:111–119 26510131 10.1001/jamasurg.2015.3637

[72] Hearn J , Higginson IJ. Development and validation of a core outcome measure for palliative care: the palliative care outcome scale. Palliative Care Core Audit Project Advisory Group. Qual Health Care 1999;8:219–227 10847883 10.1136/qshc.8.4.219 PMC2483665

[73] Turrillas P , Teixeira MJ, Maddocks M. A systematic review of training in symptom management in palliative care within postgraduate medical curriculums. J Pain Symptom Manage 2019;57:156–170.e4 30287198 10.1016/j.jpainsymman.2018.09.020

[74] Phillips AC , Lewis LK, McEvoy MP, Galipeau J, Glasziou P, Moher D et al Development and validation of the guideline for reporting evidence-based practice educational interventions and teaching (GREET). BMC Med Educ 2016;16:237 27599967 10.1186/s12909-016-0759-1 PMC5011880

[75] Van Hecke A , Duprez V, Pype P, Beeckman D, Verhaeghe S. Criteria for describing and evaluating training interventions in healthcare professions—CRe-DEPTH. Nurse Educ Today 2020;84:104254 31689586 10.1016/j.nedt.2019.104254

[76] Meinema JG , Buwalda N, van Etten-Jamaludin FS, Visser MRM, van Dijk N. Intervention descriptions in medical education: what can be improved? A systematic review and checklist. Acad Med 2019;94:281–290 30157087 10.1097/ACM.0000000000002428 PMC6365274

