# Supplementary material for: Training surgeons to optimize communication and symptom management in patients with life-limiting conditions: systematic review
Source: BJS Open. 2023 Apr 25;7(2):zrad015. doi: 10.1093/bjsopen/zrad015 (PMC10129389; doi:10.1093/bjsopen/zrad015)
Supplement: zrad015_Supplementary_Data [file zrad015_supplementary_data.zip › Supplementary_Material.docx]

**Training surgeons to optimise communication and symptom management in patients with life-limiting conditions: systematic review**

Authors: Benjamin E Zucker^1,2^, MA, MBBS; Lorna Leandro^1,2^, MA, BMBCh; Karen Forbes^1^, Ed D, MB ChB; Jane M Blazeby^1,3^, MBChB, MSc, MD; Charlotte Chamberlain^1,2^, MBBS, MSc, PhD

_1. University of Bristol, Population Health Sciences_

_2. University Hospitals Bristol NHS Foundation Trust_

_3. NIHR Biomedical Research Centre, University Hospitals Bristol and Weston NHS Foundation Trust and the University of Bristol_

**Corresponding author.** Mr Benjamin Zucker, Centre for Surgical research, Canynge Hall University of Bristol, BS8 2PL. Email: qo19163@bristol.ac.uk

**Supplementary Materials - Index**

| **Supplementary Appendixes** |  |
| --- | --- |
| Appendix S1. Search Strategy | *pag. 2* |
| **Supplementary Figures and Tables** |  |
| Table S1. Summary table of included studies  Table S2. Risk of bias | *pag. 8*  *pag. 17* |
|  |  |

**Supplementary Appendixes**

Appendix S1. Search strategy: Medline, Embase, Amed via Ovid SP

| 1 | Terminal Care.sh. | |
| --- | --- | --- |
| 2 | Terminal Care*.af. | |
| 3 | Advance Care Planning.sh. | |
| 4 | Hospices.sh. | |
| 5 | Palliative Care.sh. | |
| 6 | Palliative Medicine.sh. | |
| 7 | Terminal care*.tw. | |
| 8 | terminal illness*.tw. | |
| 9 | advance care plan*.tw. | |
| 10 | living will*.tw. | |
| 11 | end of life*.tw. | |
| 12 | advance directive*.tw. | |
| 13 | do-not-resuscitate*.tw. | |
| 14 | comfort measure*.tw. | |
| 15 | code status*.tw. | |
| 16 | palliat*.tw. |  |
| 17 | hospice*.tw. | |
| 18 | symptom management.sh. | |
| 19 | Patient care planning.sh. | |
| 20 | Ethics, Medical*.sh. | |
| 21 | Advance disease*.tw. | |
| 22 | Advance illness*.tw. | |
| 23 | Advance cancer*.tw. | |
| 24 | end stage*.tw. | |
| 25 | dying.tw. |  |
| 26 | physician-patient communication.tw. | |
| 27 | (Hospice and Palliative Care Nursing).tw. | |
| 28 | symptom management.tw. | |
| 29 | communication skils.tw. | |
| 30 | physician-patient communication.af. | |
| 31 | Surgeons.sh. | |
| 32 | general surgery.sh. | |
| 33 | surgeon*.tw. | |
| 34 | surgical*.tw. | |
| 35 | surgery*.tw. | |
| 36 | Anesthesia Department, Hospital.sh. | |
| 37 | Anesthesiology.sh. | |
| 38 | anesthesia*.tw. | |
| 39 | anaesthesia*.tw. | |
| 40 | anesthesiology*.tw. | |
| 41 | anaesthesiology*.tw. | |
| 42 | anesthesiologist*.tw. | |
| 43 | anaesthesiologist*.tw. | |
| 44 | resident.tw. | |
| 45 | residents.tw. | |
| 46 | trainee*.tw. |  |
| 47 | (Internship and residency).tw. | |
| 48 | Surgery residents.tw. | |
| 49 | intensivist*.tw. | |
| 50 | intensivists*.tw. | |
| 51 | gynaecologist*.tw. | |
| 52 | gynaecologists*.tw. | |
| 53 | critical care.sh. | |
| 54 | gynecologist*.tw. | |
| 55 | gynecologic surgical procedures.sh. | |
| 56 | Education.sh. | |
| 57 | educat* interven*.tw. | |
| 58 | Communication.sh. | |
| 59 | teaching materials.sh. | |
| 60 | Education, Medical*.sh. | |
| 61 | teaching.sh. | |
| 62 | postgraduate education*.tw. | |
| 63 | Health plan implementation.sh. | |
| 64 | Surgical training.sh. | |
| 65 | surgeon training.tw. | |
| 66 | General Surgery education*.tw. | |
| 67 | surgeon training.af. | |
| 68 | implementation.af. | |
| 69 | Surgical education.tw. | |
| 70 | 1 or 2 or 3 or 4 or 5 or 6 or 7 or 8 or 9 or 10 or 11 or 12 or 13 or 14 or 15 or 16 or 17 or 18 or 19 or 20 or 21 or 22 or 23 or 24 or 25 or 26 or 27 or 28 or 29 **or 30** | |
| 71 | 31 or 32 or 33 or 34 or 35 or 36 or 37 or 38 or 39 or 40 or 41 or 42 or 43 or 44 or 45 or 46 or 47 or 48 or 49 or 50 or 51 or 52 or 53 or 54 or 55 | |
| 72 | 56 or 57 or 58 or 59 or 60 or 61 or 62 or 63 or 64 or 65 or 66 or 67 or 68 or 69 | |
| 73 | 70 AND 71 AND 72 | |

Search strategy: Cochrane central trials register

| 1. MeSH descriptor: [Terminal Care] explode all trees |
| --- |
| 1. (Terminal care*) |
| 1. MeSH descriptor: [Advance Care Planning] explode all trees |
| 1. MeSH descriptor: [Hospices] explode all trees |
| 1. MeSH descriptor: [Palliative Care] explode all trees |
| 1. MeSH descriptor: [Palliative Medicine] explode all trees |
| 1. (terminal care*):ti,ab,kw |
| 1. (terminal illness*):ti,ab,kw |
| 1. (advance care plan*):ti,ab,kw |
| 1. (living will*):ti,ab,kw |
| 1. (end of life*):ti,ab,kw |
| 1. (advance directive*):ti,ab,kw |
| 1. (do not resuscitate*):ti,ab,kw |
| 1. (comfort measure*):ti,ab,kw |
| 1. (code status*):ti,ab,kw |
| 1. (palliat*):ti,ab,kw |
| 1. (hospice*):ti,ab,kw |
| 1. MeSH descriptor: [Symptom Assessment] explode all trees |
| 1. MeSH descriptor: [Patient Care Planning] explode all trees |
| 1. MeSH descriptor: [Ethics, Medical] explode all trees |
| 1. (advance disease*):ti,ab,kw |
| 1. (advance illness*):ti,ab,kw |
| 1. (advance cancer*):ti,ab,kw |
| 1. (end stage*):ti,ab,kw |
| 1. (dying):ti,ab,kw |
| 1. MeSH descriptor: [Surgeons] explode all trees |
| 1. MeSH descriptor: [General Surgery] explode all trees |
| 1. (surgeon*):ti,ab,kw |
| 1. (surgical*):ti,ab,kw |
| 1. (surgery*):ti,ab,kw |
| 1. MeSH descriptor: [Anesthesia Department, Hospital] explode all trees |
| 1. MeSH descriptor: [Anesthesiology] explode all trees |
| 1. (anesthesia*):ti,ab,kw |
| 1. (Anaesthesia*):ti,ab,kw |
| 1. (anesthesiology*):ti,ab,kw |
| 1. (anaesthesiology*):ti,ab,kw |
| 1. (anaesthesiologist*):ti,ab,kw |
| 1. (anesthesiologist*):ti,ab,kw |
| 1. (resident):ti,ab,kw |
| 1. (residents):ti,ab,kw |
| 1. (trainee*):ti,ab,kw |
| 1. MeSH descriptor: [Education] explode all trees |
| 1. (educat* interven*):ti,ab,kw |
| 1. MeSH descriptor: [Communication] explode all trees |
| 1. MeSH descriptor: [Teaching Materials] explode all trees |
| 1. MeSH descriptor: [Education, Medical] explode all trees |
| 1. MeSH descriptor: [Teaching] explode all trees |
| 1. (postgraduate education*):ti,ab,kw |
| 1. MeSH descriptor: [Health Plan Implementation] explode all trees |
| 1. (surgical training):ti,ab,kw |
| 1. (surgical education):ti,ab,kw |
| 1. (surgery residents):ti,ab,kw |
| 1. (surgeon training):ti,ab,kw |
| 1. (physician-patient communication):ti,ab,kw |
| 1. (Hospice and Palliative Care Nursing):ti,ab,kw |
| 1. (internship and residency):ti,ab,kw |
| 1. (General Surgery education*):ti,ab,kw |
| 1. (symptom management):ti,ab,kw |
| 1. (communication skills):ti,ab,kw |
| 1. (surgeon training) |
| 1. (physician-patient communication) |
| 1. (implementation) 2. (intensivist*):ti,ab,kw 3. (gynaecologist*):ti,ab,kw 4. ("gynaecologists"):ti,ab,kw 5. MeSH descriptor: [Gynecologic Surgical Procedures] explode all trees 6. (gynecologist*):ti,ab,kw 7. MeSH descriptor: [Critical Care] explode all trees |
| 1. 1 OR 2 OR 3 OR 4 OR 5 OR 6 OR 7 OR 8 OR 9 OR 10 OR 11 OR 12 OR 13 OR 14 OR 15 OR 16 OR 17 OR 18 OR 19 OR 20 OR 21 OR 22 OR 23 OR 24 OR 25 OR 54 OR 55 OR 58 OR 59 OR 61 |
| 1. 26 OR 27 OR 28 OR 29 OR 30 OR 31 OR 32 OR 33 OR 34 OR 35 OR 36 OR 37 OR 38 OR 39 OR 40 OR 41 OR 52 OR 56 OR 57 OR 63 OR 64 OR 65 OR 66 OR 67 OR 68 |
| 1. 42 OR 43 OR 44 OR 45 OR 46 OR 47 OR 48 OR 49 OR 50 OR 51 OR 53 OR 57 OR 60 OR 62 2. 69 AND 70 AND 71 |
|  |

**Supplementary Figures and Tables**

**Table A1: Summary table of included studies**

|  |  | Study design and rationale | | | Study participant data | | | | Data about intervention | | |
| --- | --- | --- | --- | --- | --- | --- | --- | --- | --- | --- | --- |
| Author, year | **Country** | **Study design** | **Comparison arm** | **Randomised** | **Number of participants** | **What specialty** | **Number of surgeons in intervention group?** | **Number of surgeons in control group?** | **Free text description of intervention** | **Outcome domain(s)** | **Outcomes measurement(s)** |
| Angelos et al., 1999^1^ | USA | Before-after | No | NA | 20 | General surgery residents | 20 | NA | Four 90-minute conference sessions addressing 5 topical areas: introduction, Advance directives Withdrawal or withholding treatment, DNR orders and Communicating bad news, informed consent | Communication | 1) Value: Rating of value of education 2) Knowledge: Multiple choice questions 3) Open-ended survey analysed qualitatively |
| Bergman et al., 2015^2^ | USA | Before-after | No | NA | 108 | Medical: Family medicine, internal medicine, oncology and others. Surgical: general surgery, urology and others | 43 | NA | Web-based, interactive, self-directed, case-based 15-minute learning module about end-of-life care | Communication and symptom management | 1) Attitude: Attitude questionnaire 2) Knowledge: Multiple choice questions |
| Bradley et al., 2010^3^ | USA | Cohort | Yes | No | 13 | General surgery and plastic surgery | 7 | 6 | Structured palliative care curriculum with sessions on 1) Critical appraisal of literature, 2) Breaking bad news/conducting a family meeting, 3) Patient and family support/spirituality/family presence at resuscitation 4) Ethical issues/interaction with palliative care in the surgical intensive care unit 5) Jeopardy 6) debrief | Communication and symptom management | 1) Objective skill - OSCE 2) Assessment of perceptions of palliative care - Likert scale 3) Knowledge - Core knowledge test |
| Chesney et al., 2018^4^ | Canada | Before-after | no | NA | 18 | General surgery | 18 | NA | A 2-hour intervention focused on teaching BC/WC framework including a didactic session, a live demonstration, small-group practice and debriefing | Communication | 1) Objective skill - observed standardised case  2) Acceptability of BC/WC framework - questionnaire  3) Attitudes - likert scale 4) Confidence - likert scale 5) Actions - likert scale |
| Chipman et al., 2006^5^ | USA | Single-arm experimental design | No | NA | 8 | General surgery | 8 | NA | A grand round including 45-minute lecture on EOL communication followed by Family conference OSCE with two stations: 1. EOL conversation, 2. disclosing a complication. | Communication | 1) Objective skill - OSCE 2) Value of intervention - NR how assessed |
| Fanous et al., 2017^6^ | Canada | A. Fanous+A9 | No | NA | 18 | Otolaryngology | 18 | NA | Ethical curriculum comprising didactic lectures, simulated scenarios and small group structured debriefing | Communication | 1) Objective skill - Assessment of simulated patient encounters  2) Knowledge test - Multiple choice questions  3) Perceived skill improvement - likert scale 4) Main points learned - open ended questions about main points learned |
| Gettman et al., 2008^7^ | USA | Before-after | No | NA | 19 | Urologists | 19 | NA | Simulation session of deteriorating patient including EOL conversation and breaking bad news conversation with family followed by debriefing session | Communication | 1) Objective skill - assessment of simulated situation 2) Value of experience - Likert assessment post intervention with retrospective element relating to views pre-intervention |
| Haglund et al., 2015^8^ | USA | Before-after | No | NA | 93 | Neurosurgery | 93 | NA | Multimodality intervention including a lecture, small group discussion where videos of good and bad communication in each domain were watched. Some residents were involved in role play. | Communication | 1) Value of intervention - survey questions  2) Perceived communication skills - likert scale |
| Harnof et al., 2013^9^ | Israel | Single-arm experimental design | No | NA | 15 | Neurosurgery | 15 | NA | Clinical communication and ethical dilemma simulated scenarios. Each resident participated in two scenarios and observed four. | Communication | 1) Value of intervention - Feedback questionnaire |
| Hochberg et al., 2012^10^ | USA | Interrupted time series | No | NA | 51-75 | Surgical residents | 51-75 | NA | Interactive sessions on a range of topics including: Advanced communication skills for surgical and delivering bad news | Communication | 1) Subjective professionalism skills - likert scale self  2) Objective skill - OSCE  3) Perceived change in professionalism in residents - questionnaires filled out by seniors |
| Holloran et al., 1995^11^ | USA | Serial cross sectional | No | NA | NR | Surgical residents | NR | NR | Case-based discussions for surgical residents rotating through SICU. Cases included: withholding or withdrawing treatment options, patient's treatment wishes versus the family's wishes, and incompetent patients with disagreeing family members | Communication | 1) Patient outcomes - Medical record review for evidence of patient wishes-preferences and patient-family conferences. 2) Patient outcomes - LOS of ITU patient |
| Inoue et al., 2019^12^ | Japan | Correlational | Yes | No | 923 | Respiratory physicians, pulmonary surgeons, others | 164 | 181 | Two-day workshop that provides education on the general principles of palliative care, management of pain and other major symptoms, communication skills, regional collaboration in patient care | Symptom management and communication | 1) Objective knowledge - palliative care knowledge test (PEACE-Q) 2) Self-reported practices - Palliative Care self-reported Practice Scale (PCPS)  3) Palliative care difficulties scale measured across - Palliative Care Difficulties Scale (PCDS) |
| Jameel et al., 2015^13^ | Pakistan | Before-after | No | No | 136 | Medical: General medicine, pulmonology, gastroenterology, cardiology, neurology, dermatology, haematology  Surgical: Neurosurgery, orthopaedic surgery, plastic surgery, paediatric surgery, ophthalmology Gynaecology and obstetrics Radiology | NR | NR | A 3-day workshop which started with pre-test OSCE and written test followed by group discussions on all stations and faculty presentations. This was followed by post-test using same stations | Communication | 1) Objective skill - OSCE and Written case scenario 2) Value of intervention - Likert scale |
| Klaristenfeld et al., 2007^14^ | USA | Before-after | No | No | 47 | Surgical residents' | 47 | NA | Three weekly sessions in which residents reviewed palliative care literature and performed role-plying exercises | Communication | 1) Objective knowledge - informational questionnaire 2) Value of intervention - Feedback questionnaire 3) Subjective comfort - informational and situational comfort questionnaire 4) perceived value of palliative care on surgical education - surgical training questionnaire |
| Kruser et al., 2017^15^ | USA | Single-arm experimental design | No | No | 25 | General surgery, vascular surgery, cardiac surgery, thoracic surgery | 25 | NA | A two-hour training session including a 15 min lecture followed by demonstration. The surgeon then had two cases with which to practice. Intervention was one on one with feedback throughout. | Communication | 1) Objective skill - surgeon conversation transcript assessment 2) Value of intervention - survey 3) Patient outcomes - Patient/family evaluation of BC/WC |
| Margolis et al., 2018^16^ | USA | Before-after | No | No | 20 | OB/GYN | 20 | NA | Intervention comprised: journal article review, 1-hour interactive online module, 90-minute workshop including didactic teaching and role play. 20-minute videotaped simulated patient encounter | Communication | 1) Objective skill - Evaluation of role play, evaluation of simulated patient encounter, 2) Confidence - resident self-assessment 3) Value of intervention - feedback questionnaire |
| Mikhael et al., 2008^17^ | Canada | Cluster RCT | Yes | Yes | 136 | Medicine, surgery, family medicine, other (e.g., pathology, radiation oncology, neurology, psychiatry) | 5 | 5 | Both comparison and intervention arm received two didactic sessions on EOL care. The intervention arm additionally received a pocket card with information on EOL symptom management. | Symptom management | 1) Comfort - assessed by likert scale 2) Objective knowledge - assessed by knowledge test 3) Value of intervention - assessed by qualitative interviews |
| Moon et al., 2014^18^ | USA | Before-after | No | NA | 98 | Surgical interns | 98 | NA | Facilitated discussion around breaking bad news and obtaining informed consent. Faculty members present established frameworks and session involved interaction with simulated patient with constructive feedback. | Communication (breaking bad news) | 1) Confidence - assessed by likert scale 2) Knowledge - assed by multiple choice questions 3) Value of intervention - satisfaction survey |
| Nakagawa et al., 2019^19^ | USA | Before-after | No | No | 39 | General surgery | 39 | NA | 1-hour didactic lecture on basic communication skills including NURSE statement and SPIKES protocol. Subsequently, throughout academic year residents participated in a 2-hour simulation-based communication skills practice session following vitaltalk model (skills practice session). | Communication | 1) Confidence - likert scale 2) Value - satisfaction survey 3) Self-reported actions - likert scale |
| Oya et al., 2013^20^ | Japan | Before-after | No | NA | 12 | Gastroenterological surgeons, general surgeons, pulmonary surgeon, urological surgeons, orthopaedic and plastic surgeon | 12 | NA | 1-month compulsory rotation in the palliative care department for surgical residents including treatment of patients in conjunction with palliative care team. Intervention included daily lectures | Symptom management | 1) Patient outcome - Review of patient notes for pain assessment 2) Comfort - Questionnaire 3) Questionnaire about home-based palliative care post-intervention |
| Raoof et al., 2017^21^ | USA | Before-after | No | NA | 34 | General surgery | 34 | NA | Two multimodality 55 min modules including case discussion, followed by mini-lectures related to the case and concluded with a 15-min “report-out and wrap-up” open-forum discussion. The session was completed with a 15-min were reserved for concluding remarks and evaluations/post-test. | Symptom management and communication | 1) Objective knowledge - knowledge test |
| Schmitz et al., 2016^22^ | USA | Randomised controlled trial | Yes | Y | 70 | General and orthopaedic surgery | 38 | 32 | 10 online modules (5 on error disclosure and 5 on EOL) each 10 minutes in length with additional resources and handouts. These included video clips of previously recorded OSCEs. Online teaching was paired with two 1-hour face-to-face teaching sessions | Communication | 1) Objective skill - OSCE composite score 2) Online course utilisation (exposure dosage)  3) OSCE score adjusted for 'exposure dosage' 4) Value - Feedback survey |
| Taylor et al., 2019^23^ | USA | Single-arm experimental design | No | No | 39 | General surgery, cardiothoracic surgery, vascular surgery, urology | 39 | NA | A 2-hour educational intervention including a 10 minute video on BC/WC, graphic aid practice, demonstration, role play and group discussion | Communication | 1) Objective skill - observed BC/WC simulation 2) Value of intervention - Opinion survey |
| Taylor et al., 2017^24^ | USA | Before-after | No | No | 25 | Cardiothoracic, vascular, acute care | 25 | NA | A 2-hour training session to learn the BC/WC framework using simulation with standardized patients and 1-on-1 coaching with an expert in palliative care and education | Communication | 1) Patient outcomes - including discharge destination, proportion receiving surgery 2) Objective skill - Qualitative analysis of shared decision-making consultations and Option 5 - an observer measure of shared decision making |
| Thirunavukarasu et al., 2010^25^ | USA | Before-after | No | No | 29 | General surgery | 29 | NA | Four 60minute didactic seminars, integrated with case-based discussions over 4 weeks | Communication and symptom management | 1) Perceived prevalence of ethical issues in clinical practice 2) Objective knowledge - knowledge questions 3) Confidence - confidence questions 4) Value of intervention - Attitudes toward ethics education |
| Trickey et al., 2017^26^ | USA | Interrupted time series | No | No | 12-21 | General surgery | 12-21 | NA | Quarterly communication modules (2-3 hours of dedicated classroom and practice time), with confidence evaluations for module specific objectives | Communication | 1) Objective skill - CAT item scores from simulation sessions 2) Value of intervention - perceptions of course 3) Confidence - confidence questionnaire 4) Patient outcomes - patient experience |
| Wehbe-Janek et al., 2011^27^ | USA | Before -after | No | No | 14 | General surgery | 14 | NA | 1 simulated patient encounter | Communication | 1) Objective skill - Simulated patient assessment and peer resident assessment 2) Subjective skill - self-assessment of encounter 3) Value of intervention - Resident attitudes on physician-patient communication 4) Self confidence |
| Wenger et al., 1998^28^ | USA | Non-randomised controlled trial | Yes | NR | 55 | Orthopaedic surgery | 25 | 30 | Monthly case-based didactic teaching session on: informed consent, confidentiality, privacy, truth telling, EOL decision making the physician-patient relationship, the impaired physician, and ethical issues pertaining to managed care. | Communication (EOL issues) | 1) Objective knowledge - Survey instrument  2) Value of course - Self assessment |
| Childers et al., 2018^29^ | USA | Before-after | No | No | 404 | Internal med, fam med, hospitalist, oncology, surgery. gynae onc, nephrology, physical medicine, gastro, cardio, pal care, geriatrics, critical care | 42 | NA | Four-to-eight-hour session based on the VITALTalk model, with brief lectures and demonstrations followed by practice with simulated patient cases. REMAP communication framework was used | Communication (GOC discussion) | 1) Confidence - survey assessment  2) Value of intervention - survey of satisfaction with the course 3) Patient outcomes - number of high-risk patients having GOC discussions |
| Harrington et al., 2019^30^ | USA | Before-after | No | NA | 98 | Anestesia, EM, Gen surg, plastics, vascular, unknown | 33 | NA | Experiential learning while on one-month rotation in Surgical Intensive Care | Communication | 1) Subjective confidence - Computer based survey 2) Self assessed knowledge - Computer based survey 3) Self assessed beliefs - Computer based survey |
| Minor et al., 2009^31^ | Canada | Before-after | No | NA | 17 | General surgery, internal medicine, anaesthesia, family medicine, EM, O and G | 3 | NA | Two-month rotation on mixed service ICU. Curriculum included didactic lectures, ethics seminars, and experiential learning | Both | 1) Subjective confidence - survey instrument 2) Value of intervention - survey instrument 3) Assessment of attitudes of palliative/EOL care - survey instrument |
| Brezis et al., 2017^32^ | Israel | Before-after | No | NA | 1324 | Internal medicine, geriatrics, dialysis, intensive care, neurology, neurosurgery, family medicine, EM | NR | NA | EOL simulation workshop. 6 scenarios were built: to elicit preferences for EOL care from a patient or from a relative; to handle conflicts between siblings or within the team; to handle requests to do “everything”; to explain whether to put in a feeding tube. Participants came in and received 45 min didactic lecture then proceeded to scenario followed by feedback. This cycle repeated. each participant completed 1-2 scenarios and observed several others | Communication | 1) Survey instrument 2) Qualitative assessment of feedback discussions 3) Video analysis of filmed workshops |
| Cantu et al., 2013^33^ | USA | Before-after | Yes | No | 12 | Orthopaedic surgery | 12 | NA | One-hour grand round on importance of code status and advance directive discussions. Pre-intervention and post-intervention survey of orthopaedic residents and case note review of hip fracture patients over 65 admitted over a 6-month period to identify code status discussions or ADs. | Communication | 1) Self-reported actions - Survey of frequency of CD/AD discussions and barriers to having them 2) Patient outcomes - Case not review to identify CS/AD discussions in hip fracture patients over age of 65 |
| Fiorentino et al., 2020^34^ | USA | Before-after | No | NA | 39 | EM, general surgery | 39 | NA | Pre-assessment on self-reflection on previous trauma resus' and self -assessment on perceived knowledge, comfort and confidence in holding difficult conversations in the trauma setting. Curriculum lasting one academic year including video education E-module and study guide and pocket card. The next step was a Hybrid simulated resuscitation of a high-fidelity mannequin combined with role play of delivering difficult news. Final part was OSCE of delivering news of death or poor prognosis. Each section had a post-intervention test | Communication | 1) Objective skill - assessed by OSCE 2) Subjective confidence/ comfort and knowledge - self assessment 3) Value of intervention - survey of value of educational experience 4) Assessment of team performance |
| Klingensmith et al., 2008^35^ | USA | Serial cross sectional | No | NA | NR | Compulsory for general surgery, other specialties attend sessions (Paediatric Surgery, Plastic Surgery, OBGYN, Anaesthesia, and Internal Medicine) | NR | NA | Pre-assessment was attitudinal survey for housestaff on experiences with ethical issues. Intervention included monthly case-based discussions with expert opinions. Case discussions include: when is care futile, advance directives, intent of treatment. Post-intervention assessment included attitude survey | Communication | 1) Value of intervention - Opinions and attitudes on ethics curriculum |
| Lienard et al., 2010^36^ | Belgium | Randomised controlled trial | Yes | Yes | 98 | Oncology, Gynaecology, surgery, gastro, other | NR | NR | 40-hour training program - split into bimonthly sessions over 8 months. Program was split into 30 hours of communication skills and 10hours of stress management. Communication skills: 17 hours focused on two patient communication, 10 hours on three person (i.e. patient accompanied by relative) and 3 hours on integration of communication and stress management. | Communication | 1) Objective skill - Transcripts of breaking bad news consultation analysed |
| Yamamoto et al., 2015^37^ | Japan | Before after | No | No | 223 | Internal medicine, surgery, resident, other | 51 | NA | A 2-day program with 9 modules, comprising 12-hour interactive workshops that combine didactic plenary sessions, role-play sessions, and small group discussions. | Communication and symptom management | 1) Knowledge - multiple choice test33 items across 9 domains 2) Self-reported actions - self assessment of performance of recommended practices. 3) Difficulties - Self assessment of perceived difficulties in providing palliative care |
| Perna et al.r, 2012^38^ | USA | Before after | No | No | 22 | General surgery | 22 | NA | Two-hour multi-modality workshop including didactic sessions, interactive case-based session. Four cases included which highlighted the principles behind pain control in terminally ill patients, nutritional support for terminally ill patients, conversations about code status and continuation or withdrawal of medical therapy at the end of life, and surgical interventions at the end of life. This was followed by moderated role play and completed with group discussion | Communication and symptom management | 1) Objective knowledge - Questionnaire with knowledge questions 2) Attitudes - questions assessing whether attitudes are in keeping with palliative care principles |
| Hochberg et al., 2010^39^ | USA | Before after | No | No | 15 | Surgical residents | 15 | NA | Baseline knowledge assessment through OSCE. Residents then underwent six 1-hour session training course on advanced communication skills in surgical practice: admitting mistakes, delivering bad news, interdisciplinary respect, working across language and cultural barriers, self-care. Curriculum was delivered using video vignettes, role playing, video re-enactments, role modelling, use of SPs. This was followed by another OSCE (same on as at the beginning) | Communication | 1) Objective skill - simulated patient assessment of communication and professionalism score 2) patient satisfaction |
| Larkin et al., 2010^40^ | USA | Before after | No | No | 42 | Surgical residents | 42 | NA | Aim of intervention was to improve human factors in surgeons. Participants had nine 3-hour sessions over 2 years on 1) Empathy and Caring (palliative component), 2) Time Management and Professional Development 3) Stress Management 4) Working in Teams I: The Surgical Setting 5) Informing and Educating the Patient and Family 6) Empathy and Caring II 7) Working in Teams II: Coordination of Care Across the continuum 8) Conflict resolution 9) Communicating with patients with difficult personality styles | Communication | 1) Objective skill - assessment of written responses to vignettes  2) Value of intervention - Course evaluation |
| Merckaert et al., 2013^41^ | Belgium | Randomised controlled trial | Yes | Yes | 95 | Oncology (including oncology, haematology, and radiotherapy; 6%), gynaecology (31%), and other specialties (63%). | NR | NA | 40-hour training program - split into bimonthly sessions over 8 months. Program was split into 30 hours of communication skills and 10hours of stress management. Communication skills: 17 hours focused on two patient communication, 10 hours on three person (i.e. patient accompanied by relative) and 3 hours on integration of communication and stress management. | Communication | Objective skill - analysis of transcripts of consultations |
| Zimmerman et al., 2021^42^ | USA | Before After | No | No | 43 | Trauma Surgeons | 43 | NA | Training program to teach trauma attendings and fellows to use BC/WC tool. Training included expert demonstration followed by three case practices with coaches. | Communication | 1) Patient outcome – intensity of received treatment  2) Objective skill – family and nurse assessment of communication skill  2) Value of intervention |
| Amen et al., 2021^43^ | USA | Serial cross-sectional | No | No | NR | Surgical residents and fellows | NR | NA | Multi-modal palliative care communication training intervention of one hour/week for four weeks comprising journal article review, lecture, small group discussion, role play and case review. | Communication | 1) Patient outcomes: documentation of GOC discussion, ICU LOS, ventilator days, frequency of invasive procedures, disposition and mortality |
| Weill et al., 2022^44^ | USA | Beforte After | No | No | 48 | General surgery and otolaryngology residents | 48 | NA | 2-3 hour training in best case/worst case including group discussion, vignette-based practice, role play, group discussion and didactic teaching. | Communication | 1) Confidence – self assessment using Likert scale  2) value of the intervention – assessed on Likert scale and proportion of trainees who would recommend program |
| Kaminski 2022^45^ | USA | Before After | No | No | 17 | General Surgery and advance practice providers | NR | NA | 30 minute virtual presentation including simulation videos and didactic teaching. | Communication | 1) Patient outcome - Frequency of ACP documentation  2) Confidence – self assessment using the End-of- Life Professional Caregiver Survey |
| Lockwood et al., 2022^46^ | USA | RCT | Yes | Yes | 149 | General Surgery and internal medicine residents | 22 | 22 | Eight hour VitalTalk workshop including didactic teaching, demonstration, simulation, and group discussion. Bedside feedback during clinical patient encounters. | Communication | 1) Objective skill – assessed using Family Meeting Behavioral Skills Checklist during simulated patient encounters  2) Confidence – self assessment |

**Table S2: Risk of bias table**

| **Author, year** | **Cohort study** | **Control/comparison group** | **Pre/post intervention data** | **Random assignment of participants** | **Random selection of participants for assessment** | **Follow-up rate of 80% or more** | **Comparison groups equivalent on sociodemographics** | **Comparison groups equivalent at baseline on outcome measures** |
| --- | --- | --- | --- | --- | --- | --- | --- | --- |
| Angelos et al., 1999^1^ | Yes | No | Yes | No | No | Unclear | NA | NA |
| Bergman et al., 2015^2^ | Yes | No | Yes | NR | No | No | NA | NA |
| Bradley et al., 2010^3^ | Yes | Yes | Yes | No | No | Yes | NR | Yes |
| Chesney et al., 2018^4^ | Yes | No | Yes | No | No | NR | NA | NA |
| Chipman et al., 2006^5^ | No | Yes | No | No | No | Yes | NR | NR |
| Fanous et al., 2017^6^ | Yes | No | Yes | No | No | Yes | NA | NA |
| Gettman et al., 2008^7^ | No | No | No | No | No | NA | NA | NA |
| Haglund et al., 2015^8^ | Yes | No | Yes | No | No | Yes | NA | NA |
| Harnof et al., 2013^9^ | No | No | No | No | No | Unclear | NA | NA |
| Hochberg et al., 2012^10^ | Yes | No | Yes | No | No | No | NA | NA |
| Holloran et al., 1995^11^ | No | No | No | No | No | NA | NA | NA |
| Inoue et al., 2019^12^ | No | Yes | No | No | No | NA | No | NA |
| Jameel et al., 2015^13^ | Yes | No | Yes | No | No | Yes | NA | NA |
| Klaristenfeld et al., 2007^14^ | Yes | No | Yes | No | No | Yes | NA | NA |
| Kruser et al., 2017^15^ | Yes | No | No | No | No | Yes | NA | NA |
| Margolis et al., 2018^16^ | Yes | No | Yes | No | No | No | NA | NA |
| Mikhael et al., 2008^17^ | Yes | Yes | Yes | Yes | No | Yes | Yes | No |
| Moon et al., 2014^18^ | Yes | No | Yes | No | No | Yes | NA | NA |
| Nakagawa et al., 2019^19^ | Yes | No | Yes | No | No | Yes | NA | NA |
| Oya et al., 2013^20^ | Yes | No | Yes | No | No | Yes | NA | NA |
| Raoof et al., 2017^21^ | Yes | No | Yes | No | No | No | NA | NA |
| Schmitz et al., 2016^22^ | Yes | Yes | Yes | Yes | No | No | NR | Yes |
| Taylor et al., 2019^23^ | Yes | No | No | No | No | No | NA | NA |
| Taylor et al., 2017^24^ | Yes | No | Yes | No | No | Yes | NA | NA |
| Thirunavukarasu et al., 2010^25^ | Yes | No | Yes | No | No | No | NA | NA |
| Trickey et al., 2017^26^ | Yes | No | Yes | No | No | No | NA | NA |
| Wehbe-Janek et al., 2011^27^ | Yes | No | Yes | NA | No | Unclear | NA | NA |
| Wenger et al., 1998^28^ | Yes | Yes | Yes | No | No | Yes | Yes | Yes |
| Childers et al., 2018^29^ | Yes | No | Yes | No | No | Yes | NA | NA |
| Harrington et al., 2019^30^ | Yes | No | Yes | No | No | Yes | NA | NA |
| Minor et al., 2009^31^ | Yes | No | Yes | No | No | Yes | NA | NA |
| Brezis et al., 2017^32^ | Yes | No | Yes | NA | No | Yes | NA | NA |
| Cantu et al., 2013^33^ | Yes | Yes | Yes | No | No | NR | NA | NA |
| Fiorentino et al., 2020^34^ | Yes | No | Yes | No | No | Yes | NA | NA |
| Klingensmith et al., 2008^35^ | No | No | Yes | No | No | NR | NA | NA |
| Lienard et al., 2010^36^ | Yes | Yes | Yes | Yes | No | Yes | No | NR |
| Yamamoto et al., 2015^37^ | Yes | No | Yes | No | No | Yes | NA | NA |
| Perna et al.r, 2012^38^ | Yes | No | Yes | No | No | No | NA | NA |
| Hochberg et al., 2010^39^ | Yes | No | Yes | NA | No | No | NA | NA |
| Larkin et al., 2010^40^ | Yes | No | Yes | No | No | No | NA | NA |
| Merckaert et al., 2013^41^ | Yes | Yes | Yes | Yes | No | Yes | Yes | NR |
| Zimmerman et al., 2021^42^ | Yes | No | Yes | No | No | NR | NA | NA |
| Amen et al., 2021^43^ | No | No | Yes | No | No | NA | NA | NA |
| Weill et al., 2022^44^ | Yes | No | Yes | No | No | Yes | NA | NA |
| Kaminski 2022 | Yes | No | Yes | No | No | No | NA | NA |
| Lockwood et al., 2022^46^ | Yes | Yes | Yes | Yes | No | No | NR | Yes |

**References**

1. Angelos P, DaRosa DA, Derossis AM, Kim B. Medical ethics curriculum for surgical residents: Results of a pilot project. *Surgery*. 1999;126(4):701-707. doi:<http://dx.doi.org/10.1016/S0039-6060%2899%2970125-X>

2. Bergman J, Lorenz KA, Ballon-Landa E, et al. A Scalable Web-Based Module for Improving Surgical and Medical Practitioner Knowledge and Attitudes about Palliative and End-of-Life Care. *Journal of palliative medicine*. 2015;18(5):415-20. doi:<https://dx.doi.org/10.1089/jpm.2014.0349>

3. Bradley CT, Webb TP, Schmitz CC, Chipman JG, Brasel KJ. Structured teaching versus experiential learning of palliative care for surgical residents. *American journal of surgery*. 2010;200(4):542-7. doi:<https://dx.doi.org/10.1016/j.amjsurg.2009.12.014>

4. Chesney T, Devon K. Training surgical residents to use a framework to promote shared decision-making for patients with poor prognosis experiencing surgical emergencies. *Canadian journal of surgery Journal canadien de chirurgie*. 2018;61(2):114-120.

5. Chipman JG, Beilman GJ, Schmitz CC, Seatter SC. Development and pilot testing of an OSCE for difficult conversations in surgical intensive care. *Journal of surgical education*. 2007;64(2):79-87.

6. Fanous A, Rappaport J, Young M, Park YS, Manoukian J, Nguyen LHP. A longitudinal simulation-based ethical-legal curriculum for otolaryngology residents. *The Laryngoscope*. 2017;127(11):2501-2509. doi:<https://dx.doi.org/10.1002/lary.26551>

7. Gettman MT, Karnes RJ, Arnold JJ, et al. Urology resident training with an unexpected patient death scenario: experiential learning with high fidelity simulation. *The Journal of urology*. 2008;180(1):283-288. doi:<https://dx.doi.org/10.1016/j.juro.2008.03.042>

8. Haglund MM, Rudd M, Nagler A, Prose NS. Difficult conversations: a national course for neurosurgery residents in physician-patient communication. *Journal of surgical education*. 2015;72(3):394-401. doi:<https://dx.doi.org/10.1016/j.jsurg.2014.11.014>

9. Harnof S, Hadani M, Ziv A, Berkenstadt H. Simulation-based interpersonal communication skills training for neurosurgical residents. *The Israel Medical Association journal : IMAJ*. 2013;15(9):489-92.

10. Hochberg MS, Berman RS, Kalet AL, et al. The professionalism curriculum as a cultural change agent in surgical residency education. *American journal of surgery*. 2012;203(1):14-20. doi:<https://dx.doi.org/10.1016/j.amjsurg.2011.05.007>

11. Holloran SD, Starkey GW, Burke PA, et al. An educational intervention in the surgical intensive care unit to improve ethical decisions. *Surgery*. 1995;118(2):294-299. doi:<http://dx.doi.org/10.1016/S0039-6060%2805%2980337-X>

12. Inoue A, Yamaguchi T, Tanaka K, et al. Benefits of a Nationwide Palliative Care Education Program on Lung Cancer Physicians. *Internal medicine (Tokyo, Japan)*. 2019;58(10):1399-1403. doi:<https://dx.doi.org/10.2169/internalmedicine.0872-18>

13. Jameel A, Noor SM, Ayub S, Ali SS, Park YS, Tekian A. Feasibility, relevance and effectiveness of teaching and assessment of ethical status and communication skills as attributes of professionalism. *JPMA The Journal of the Pakistan Medical Association*. 2015;65(7):721-6.

14. Klaristenfeld DD, Harrington DT, Miner TJ. Teaching palliative care and end-of-life issues: a core curriculum for surgical residents. *Annals of surgical oncology*. 2007;14(6):1801-6.

15. Kruser JM, Taylor LJ, Campbell TC, et al. "Best Case/Worst Case": Training Surgeons to Use a Novel Communication Tool for High-Risk Acute Surgical Problems. *Journal of pain and symptom management*. 2017;53(4):711-719.e5. doi:<https://dx.doi.org/10.1016/j.jpainsymman.2016.11.014>

16. Margolis B, Blinderman C, de Meritens AB, et al. Educational Intervention to Improve Code Status Discussion Proficiency Among Obstetrics and Gynecology Residents. *The American journal of hospice & palliative care*. 2018;35(4):724-730. doi:<https://dx.doi.org/10.1177/1049909117733436>

17. Mikhael J, Baker L, Downar J. Using a pocket card to improve end-of-life care on internal medicine clinical teaching units: a cluster-randomized controlled trial. *Journal of general internal medicine*. 2008;23(8):1222-7. doi:<https://dx.doi.org/10.1007/s11606-008-0582-4>

18. Moon MR, Hughes MT, Chen J-Y, Khaira K, Lipsett P, Carrese JA. Ethics skills laboratory experience for surgery interns. *Journal of surgical education*. 2014;71(6):829-38. doi:<https://dx.doi.org/10.1016/j.jsurg.2014.03.010>

19. Nakagawa S, Fischkoff K, Berlin A, Arnell TD, Blinderman CD. Communication Skills Training for General Surgery Residents. *Journal of surgical education*. 2019;76(5):1223-1230. doi:<https://dx.doi.org/10.1016/j.jsurg.2019.04.001>

20. Oya H, Matoba M, Murakami S, et al. Mandatory palliative care education for surgical residents: initial focus on teaching pain management. *Japanese journal of clinical oncology*. 2013;43(2):170-5. doi:<https://dx.doi.org/10.1093/jjco/hys205>

21. Raoof M, O'Neill L, Neumayer L, Fain M, Krouse R. Prospective evaluation of surgical palliative care immersion training for general surgery residents. *American Journal of Surgery*. 2017;214(2):378-383. doi:<http://dx.doi.org/10.1016/j.amjsurg.2016.11.032>

22. Schmitz CC, Braman JP, Turner N, et al. Learning by (video) example: a randomized study of communication skills training for end-of-life and error disclosure family care conferences. *American journal of surgery*. 2016;212(5):996-1004. doi:<https://dx.doi.org/10.1016/j.amjsurg.2016.02.023>

23. Taylor LJ, Adkins S, Hoel AW, et al. Using Implementation Science to Adapt a Training Program to Assist Surgeons with High-Stakes Communication. *Journal of surgical education*. 2019;76(1):165-173. doi:<https://dx.doi.org/10.1016/j.jsurg.2018.05.015>

24. Taylor LJ, Nabozny MJ, Steffens NM, et al. A Framework to Improve Surgeon Communication in High-Stakes Surgical Decisions: Best Case/Worst Case. *JAMA surgery*. 2017;152(6):531-538. [Comment in: JAMA Surg. 2017 Jun 1;152(6):538-539; PMID: 28146226 [<https://www.ncbi.nlm.nih.gov/pubmed/28146226>]]. doi:<https://dx.doi.org/10.1001/jamasurg.2016.5674>

25. Thirunavukarasu P, Brewster LP, Pecora SM, Hall DE. Educational intervention is effective in improving knowledge and confidence in surgical ethics-a prospective study. *American journal of surgery*. 2010;200(5):665-9. doi:<https://dx.doi.org/10.1016/j.amjsurg.2010.08.002>

26. Trickey AW, Newcomb AB, Porrey M, et al. Two-Year Experience Implementing a Curriculum to Improve Residents' Patient-Centered Communication Skills. *Journal of surgical education*. 2017;74(6):e124-e132. doi:<https://dx.doi.org/10.1016/j.jsurg.2017.07.014>

27. Wehbe-Janek H, Song J, Shabahang M. An evaluation of the usefulness of the standardized patient methodology in the assessment of surgery residents' communication skills. *Journal of surgical education*. 2011;68(3):172-7. doi:<https://dx.doi.org/10.1016/j.jsurg.2010.12.005>

28. Wenger NS, Liu H, Lieberman JR. Teaching medical ethics to orthopaedic surgery residents. *The Journal of bone and joint surgery American volume*. 1998;80(8):1125-31.

29. Childers JW, Arnold RM. Expanding Goals of Care Conversations Across a Health System: The Mapping the Future Program. *J Pain Symptom Manage*. Oct 2018;56(4):637-644. doi:10.1016/j.jpainsymman.2018.07.013

30. Harrington AW, Oliveira KD, Lui FY, Maerz LL. Resident Education in End-of-Life Communication and Management: Assessing Comfort Level to Enhance Competence and Confidence. *Journal of surgical education*. 2019;doi:<http://dx.doi.org/10.1016/j.jsurg.2019.11.003>

31. Minor S, Schroder C, Heyland D. Using the intensive care unit to teach end-of-life skills to rotating junior residents. *American Journal of Surgery*. 2009;197(6):814-819. doi:<http://dx.doi.org/10.1016/j.amjsurg.2008.04.015>

32. Brezis M, Lahat Y, Frankel M, et al. What can we learn from simulation-based training to improve skills for end-of-life care? Insights from a national project in Israel. *Israel journal of health policy research*. 2017;6(1):48. [Comment in: Isr J Health Policy Res. 2017 Nov 20;6(1):62; PMID: 29157290 [<https://www.ncbi.nlm.nih.gov/pubmed/29157290>]]. doi:<https://dx.doi.org/10.1186/s13584-017-0169-9>

33. Cantu RV, Coe MP, Pober DM, Byock IR. Orthopedic grand rounds can change resident practice. *American journal of orthopedics (Belle Mead, NJ)*. 2013;42(5):215-219.

34. Fiorentino M, Mosenthal AC, Bryczkowski S, Lamba S. Teaching Residents Communication Skills around Death and Dying in the Trauma Bay. *Journal of palliative medicine*. 2020;doi:<http://dx.doi.org/10.1089/jpm.2020.0076>

35. Klingensmith ME. Teaching ethics in surgical training programs using a case-based format. *Journal of surgical education*. 2008;65(2):126-8. doi:<https://dx.doi.org/10.1016/j.jsurg.2007.12.001>

36. Liénard A, Merckaert I, Libert Y, et al. Is it possible to improve residents breaking bad news skills? A randomised study assessing the efficacy of a communication skills training program. *Br J Cancer*. Jul 13 2010;103(2):171-7. doi:10.1038/sj.bjc.6605749

37. Yamamoto R, Kizawa Y, Nakazawa Y, Ohde S, Tetsumi S, Miyashita M. Outcome evaluation of the Palliative care Emphasis program on symptom management and Assessment for Continuous Medical Education: nationwide physician education project for primary palliative care in Japan. *J Palliat Med*. Jan 2015;18(1):45-9. doi:10.1089/jpm.2014.0122

38. Pernar LIM, Peyre SE, Smink DS, Block SD, Cooper ZR. Feasibility and Impact of a Case-Based Palliative Care Workshop for General Surgery Residents. *Journal of the American College of Surgeons*. doi:<http://dx.doi.org/10.1016/j.jamcollsurg.2011.11.002>

39. Hochberg MS, Kalet A, Zabar S, Kachur E, Gillespie C, Berman RS. Can professionalism be taught? Encouraging evidence. *Am J Surg*. Jan 2010;199(1):86-93. doi:10.1016/j.amjsurg.2009.10.002

40. Larkin AC, Cahan MA, Whalen G, et al. Human Emotion and Response in Surgery (HEARS): a simulation-based curriculum for communication skills, systems-based practice, and professionalism in surgical residency training. *J Am Coll Surg*. Aug 2010;211(2):285-92. doi:10.1016/j.jamcollsurg.2010.04.004

41. Merckaert I, Liénard A, Libert Y, et al. Is it possible to improve the breaking bad news skills of residents when a relative is present? A randomised study. *Br J Cancer*. Nov 12 2013;109(10):2507-14. doi:10.1038/bjc.2013.615

42. Zimmermann CJ, Zelenski AB, Buffington A, et al. Best case/worst case for the trauma ICU: Development and pilot testing of a communication tool for older adults with traumatic injury. *Journal of Trauma and Acute Care Surgery*. 2021;91(3):542-551. doi:<https://dx.doi.org/10.1097/TA.0000000000003281>

43. Amen SS, Berndtson AE, Cain J, et al. Communication and Palliation in Trauma Critical Care: Impact of Trainee Education and Mentorship. *The Journal of surgical research*. 2021;266:236-244. doi:<https://dx.doi.org/10.1016/j.jss.2021.03.005>

44. Weill SR, Layden AJ, Nabozny MJ, et al. Applying VitalTalkTM Techniques to Best Case/Worst Case Training to Increase Scalability and Improve Surgeon Confidence in Shared Decision-making. *Journal of surgical education*. 2022;79(4):983-992. doi:<https://dx.doi.org/10.1016/j.jsurg.2022.01.012>

45. Kaminski A. Let's Talk About Dying: An Educational Pilot Program to Improve Providers' Competency in End-Of-Life Discussions. *The American journal of hospice & palliative care*. 2022:10499091221127994. doi:<https://dx.doi.org/10.1177/10499091221127994>

46. Lockwood BJ, Gustin J, Verbeck N, et al. Training to Promote Empathic Communication in Graduate Medical Education: A Shared Learning Intervention in Internal Medicine and General Surgery. *Palliative medicine reports*. 2022;3(1):26-35. doi:<https://dx.doi.org/10.1089/pmr.2021.0036>
